# A systematic review and synthesis of global stroke guidelines on behalf of the World Stroke Organization

**DOI:** 10.1177/17474930231156753

**Published:** 2023-03-01

**Authors:** Gillian E Mead, Luciano A Sposato, Gisele Sampaio Silva, Laetitia Yperzeele, Simiao Wu, Mansur Kutlubaev, Joshua Cheyne, Kolawole Wahab, Victor C Urrutia, Vijay K Sharma, PN Sylaja, Kelvin Hill, Thorsten Steiner, David S Liebeskind, Alejandro A Rabinstein

**Affiliations:** 1 Usher Institute, University of Edinburgh and Royal Infirmary of Edinburgh, Little France Crescent, Edinburgh, UK; 2 Department of Clinical Neurological Sciences, London Health Sciences Centre, Western University, London, ON, Canada; 3 Heart & Brain Lab, Western University, London, ON, Canada; 4 Robarts Research Institute, London, ON, Canada; 5 Lawson Health Research Institute, London, ON, Canada; 6 Department of Neurology and Neurosurgery, Federal University of São Paulo (UNIFESP), São Paulo, Brazil; 7 Hospital Israelita Albert Einstein, São Paulo, Brazil; 8 Antwerp NeuroVascular Center and Stroke Unit, Antwerp University Hospital, Antwerp, Belgium; 9 Research Group on Translational Neurosciences, Faculty of Medicine and Health Sciences, University of Antwerp, Antwerp, Belgium; 10 Department of Neurology, West China Hospital, Sichuan University, Chengdu, China; 11 Department of Neurology, Bashkir State Medical University, Ufa, Russia; 12 Centre for Clinical Brain Sciences, University of Edinburgh, Edinburgh, UK; 13 Department of Medicine, University of Ilorin, Ilorin, Nigeria; 14 Department of Neurology, Johns Hopkins University School of Medicine, Baltimore, MD, USA; 15 Yong Loo Lin School of Medicine, National University of Singapore, Singapore; 16 Division of Neurology, University Medicine Cluster, National University Health System, Singapore; 17 Neurology and Comprehensive Stroke Care Program, Sree Chitra Tirunal Institute for Medical Sciences and Technology, Thiruvananthapuram, India; 18 Stroke Treatment, Stroke Foundation, Melbourne, VIC, Australia; 19 Departments of Neurology, Klinikum Frankfurt Höchst and Heidelberg University Hospital, Frankfurt, Germany; 20 UCLA Department of Neurology, Neurovascular Imaging Research Core, UCLA Comprehensive Stroke Center, Los Angeles, CA, USA; 21 Neurology, Mayo Clinic, Rochester, MN, USA

**Keywords:** Stroke rehabilitation, stroke care, guidelines, acute stroke care, secondary stroke prevention

## Abstract

**Background:**

There are multiple stroke guidelines globally. To synthesize these and summarize what existing stroke guidelines recommend about the management of people with stroke, the World Stroke Organization (WSO) Guideline committee, under the auspices of the WSO, reviewed available guidelines.

**Aims:**

To systematically review the literature to identify stroke guidelines (excluding primary stroke prevention and subarachnoid hemorrhage) since 1 January 2011, evaluate quality (The international Appraisal of Guidelines, Research and Evaluation (AGREE II)), tabulate strong recommendations, and judge applicability according to stroke care available (minimal, essential, advanced).

**Summary of review:**

Searches identified 15,400 titles; 911 texts were retrieved, 200 publications scrutinized by the three subgroups (acute, secondary prevention, rehabilitation), and recommendations extracted from most recent version of relevant guidelines. For acute treatment, there were more guidelines about ischemic stroke than intracerebral hemorrhage; recommendations addressed pre-hospital, emergency, and acute hospital care. Strong recommendations were made for reperfusion therapies for acute ischemic stroke. For secondary prevention, strong recommendations included establishing etiological diagnosis; management of hypertension, weight, diabetes, lipids, and lifestyle modification; and for ischemic stroke, management of atrial fibrillation, valvular heart disease, left ventricular and atrial thrombi, patent foramen ovale, atherosclerotic extracranial large vessel disease, intracranial atherosclerotic disease, and antithrombotics in non-cardioembolic stroke. For rehabilitation, there were strong recommendations for organized stroke unit care, multidisciplinary rehabilitation, task-specific training, fitness training, and specific interventions for post-stroke impairments. Most recommendations were from high-income countries, and most did not consider comorbidity, resource implications, and implementation. Patient and public involvement was limited.

**Conclusion:**

The review identified a number of areas of stroke care where there was strong consensus. However, there was extensive repetition and redundancy in guideline recommendations. Future guideline groups should consider closer collaboration to improve efficiency, include more people with lived experience in the development process, consider comorbidity, and advise on implementation.

## Background

The World Stroke Organization’s (WSO) mission is to reduce the global burden of stroke through prevention, treatment, and long-term care. In 2014, Lindsay et al. produced a Global Stroke Services Action plan,^1^ based on recommendations from the 10 stroke guidelines which achieved scores of greater than 60% on two (Rigor and Editorial Independence) of the six domains from The international Appraisal of Guidelines, Research and Evaluation (AGREE II) tool. The authors judged the applicability of recommendations in minimal, essential, and advanced stroke services.

In 2021, the WSO Board tasked the existing Stroke Guidelines and Quality Committee (the Committee) to update the synthesis of global guidelines. The Committee included one lay member. With the approval from the WSO Board, additional co-opted members with expertise in systematic reviews and guideline production were added by open competition, balanced by gender, seniority, and location. All members provided conflict of interest statements prior to publication. No funding was provided for this work. It was agreed that the work would be published in *International Journal of Stroke* after peer review.

## Aims

Our broad question was “What do existing stroke guidelines recommend about the management of people with stroke?” Our specific objectives are described in our PROSPERO protocol (CRD42021268434, 26 July 2022). Based on the recommendations, the group devised metrics for service audit.

## Methods

Literature searches were devised and run by Joshua Cheyne, Information Specialist of Cochrane Stroke (Supplemental Appendix 1).

### Participants/population

**Inclusion criteria:** Stroke, as defined by the American Heart Association,^2^ not subarachnoid hemorrhage. We did not specifically seek papers for patients with transient ischemic attack (TIA), but where the guidelines included recommendations for TIA, these were extracted too. This mainly applied to the secondary prevention guidelines.

**Intervention:** Any intervention for acute care, rehabilitation, secondary prevention, but not the primary prevention of stroke. We included problems that are a direct consequence of stroke. We excluded post-stroke global cognitive impairment and dementia, as these are not solely due to stroke and to ensure our work was feasible.

**Guideline definition:** Systematically developed statements to assist practitioner and patient decisions about appropriate health care for specific clinical circumstances. Stakeholder peer review was poorly reported so we made a post hoc decision not to apply this exclusion criterion.

**Screening and selection of studies:** The literature search (performed September 2021) was imported into Covidence; titles and/or abstracts were screened by two authors, and potentially eligible full texts were reviewed by two authors who applied inclusion and exclusion criteria. We included all languages that the subgroups were fluent in, including English, Chinese, German, Spanish, Portugese, Dutch (Flemish), and French. In case of disagreement, a third person was consulted. We also scrutinized the Australian and New Zealand living guidelines, which were not identified in our searches, and extracted relevant recommendations (Clinical guidelines—Stroke Foundation—Australia).

## Collating guidelines, extracting data, and reviewing quality

There is no published methodology for collating recommendations from multiple guidelines. Thus, we used the same approach as Lindsay et al (2014), but evaluated all published guidelines since 2011. We drew heavily on our expertise in evidence syntheses.

We categorized guidelines into acute, and/or secondary prevention, and/or rehabilitation/life after stroke. Three subgroups were convened (rehabilitation: G.E.M., S.W., M.K., L.Y.; acute: A.A.R., G.S.S., T.S., V.C.U., D.S.L., P.N.S.; secondary prevention: L.A.S., K.W., K.H., V.K.S.) and each member was allocated approximately 15 guidelines each. Only “strong” recommendations were extracted. We relied on the evaluation of strength of recommendation by the authors of the individual guidelines; these recommendations were usually but not always supported by the highest grade of evidence. Sometimes, the individual guideline authors judged the recommendation to be “strong” and/or judged the evidence underpinning the recommendation to be of the highest grade, but if the writing group disagreed based on their knowledge of the literature, we noted these and did not include them in our recommendations. If a recommendation included the term “consider,” we retained the term “consider” as we did not wish to inadvertently change the meaning of the recommendation. We evaluated quality using the AGREE II tool (available at http://agreetrust.org) which has six domains (scope and purpose, stakeholder involvement, rigor of development, clarity of presentation, applicability, and editorial independence), 23 checklist items within these six domains; and some checklist items include multiple questions. Some guidelines were evaluated using AGREE II by only one reviewer due to the large number of guidelines.

“Strong” recommendations were collated into tables 1 to 3 and, where possible, very similar ones were combined to limit repetition. The group then judged applicability in advanced, essential, and minimal services (Box 1), acknowledging that these may vary between countries and within regions. We matched recommendations to level of service available—accepting that within the three categories we used, there is likely to be variation in specific interventions available (Box 1). If an intervention is indicated but not available, services should indicate this in patient’s records; which may help to drive forward service development.

**Box 1.** Definition of stroke service delivery model.^1^

Minimal level of resource availability	Essential service level	Advanced stroke services
Stroke care delivery is based at a local clinic staffed predominantly by non-physicians; laboratory tests and diagnostic studies are scarce; and much of the emphasis is placed on bedside clinical skills, teaching, and prevention	Access to a CT scan, physicians, and the potential for acute thrombolytic therapy; however, stroke expertise may still be difficult to access	Multidisciplinary stroke expertise, multimodal imaging, and comprehensive therapies are available including endovascular thrombectomy

CT: computed tomography.

A “service” may include several hospitals offering a range of stroke care.

Each group lead (G.E.M., A.A.R., L.A.S.) narratively reviewed the AGREE II forms and reported common areas of strengths and weaknesses. There is no published methodology for collating the findings of AGREE II—thus, we narratively reviewed them. These completed forms are available on request should other researchers wish to analyze the data in more detail.

Based on the recommendations, we created performance metrics for audit (Table 4). We did not use the minimal/essential/advanced categories for performance metrics because of the variation in service level within each category.

Following peer review, we removed the handful of recommendations about “what not to do.” The searches were performed in September 2021. While we could not systematically search the literature again in 2022, because this would have delayed publication of this article, we scrutinized newly published guidelines that were identified through our stakeholder consultation in November 2022. We listed the new guidelines in our reference list and checked that our recommendations were still valid. This also applied to guidelines published prior to September 2021 which had not been identified in our initial searches.

## Stakeholder involvement

In November 2022, we asked the WSO Board, including Stroke Support Organisations (which have patients as core stakeholders) to comment. In December 2022, we held a meeting with people with lived experiences (PWLE) to review an updated version of the guidelines. PWLE provided feedback at the end of the meeting based on a structured interview, including the following questions: (1) Do you think these guidelines are thorough enough to capture the most relevant aspects of stroke care? (2) Are there any aspects of stroke care that these guidelines should address? (3) Please provide a short comment about potential areas of improvement. (4) Do you have any other comments?

## Results

**Figure 1.**
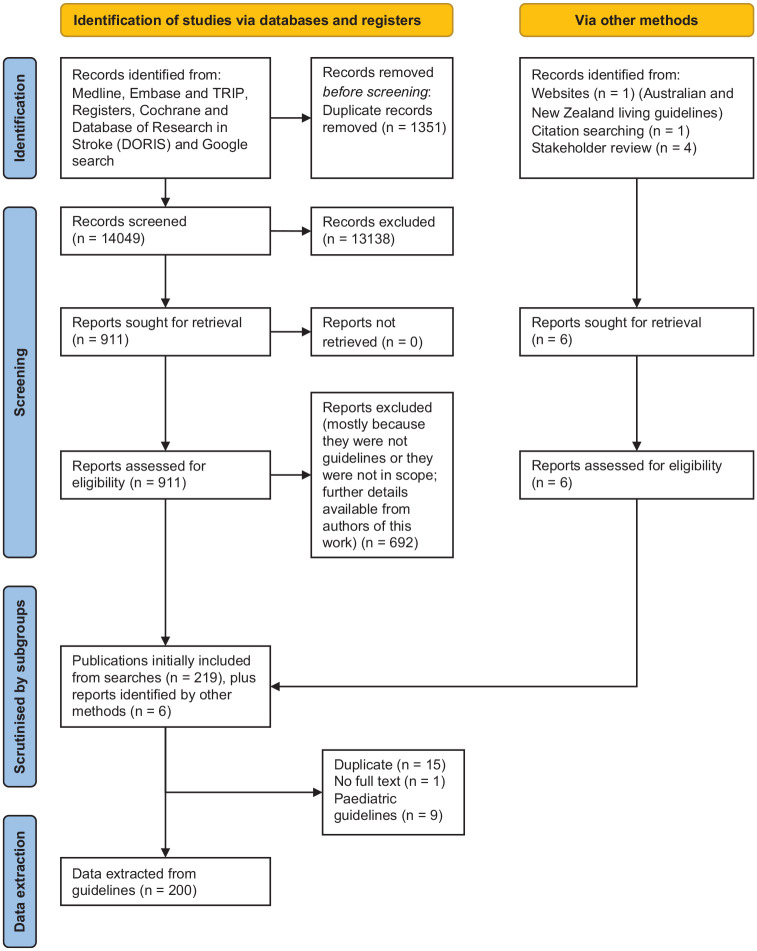
PRISMA flow diagram showing selection of guidelines.

Search results are shown in Supplemental Appendix 2 and in Figure 1. Guidelines not relevant to respective subgroups are shown in Table 3 of Supplemental Appendix. From the initial 14,049 records screened from our searches, we extracted recommendations from 200 included guidelines.^3–202^ The great majority of guidelines were from high-income countries. Fewer than a 10th covered the entire stroke pathway. Following peer review, we included five additional references to clarify secondary prevention statements.^203–207^

### Grading systems used

Different guideline groups used different grading systems. For instance, AHA used Classes I, II, and III, with associated levels of evidence A–C; Department of Veterans Affairs/Department of Defense (VA/DoD) used “strong/weak,” either for or against; European Stroke Organization (ESO) and Australia and New Zealand Guideline group uses strength of recommendations divided into four categories (strong or weak for or against) based PICO (patient, intervention, comparison, outcome) questions according to the GRADE approach); and Canadian guidelines used A or B or C for strength of recommendations. National Institute of Clinical Excellence (NICE) and Royal College of Physicians of London (RCPL) provide just the recommendations; in the absence of any grading, we assumed all recommendations were strong and included them.

### Quality assessments by AGREE II

Our quality assessments showed that in general, the objectives, populations, questions (aspects 1–3 of AGREE II), group membership and target users were defined but the target population, preferences, and views were not so clearly described. There was a lack of patient, public and caregiver authorship of guidelines.

Very few guidelines set out to consider a priori the impact of comorbidity on recommendations, for example, interaction between treatment for hypertension and diabetes in the same person, interaction between atrial fibrillation, smoking, and diabetes; the impact of frailty and other conditions such as dementia; and how polypharmacy prior to stroke might influence treatment options. The guideline developers may have known in advance that the majority of primary research studies do not consider these interactions. Search methods and evidence selection criteria were generally well described, and strength and limitations of guidelines were satisfactory. Methods for external review were generally not well described, and most did not provide the date for a planned update.

Recommendations were generally specific and unambiguous, though the word “considered” was often used for “strong recommendations,” implying some uncertainty in the evidence. Future guidelines groups should clarify the meaning of “consider.” Management options were generally described well. Recommendations were listed either in a table (which were quick to extract) or in the text.

Facilitators and barriers to application were generally not discussed. Only some of the bigger groups provided implementation tools and advice. Resource implications were generally not discussed.

The funding body was generally recorded but some guidelines did not specifically state whether the funder had an influence on the guidelines. Competing interests were generally given for each author, but how these interests influenced the conclusions of the guidelines was not so well reported. Patient and public involvement (PPI) was limited, and stakeholder peer review was infrequently reported.

The detailed recommendations for each of the three subgroups (acute, rehabilitation, and secondary prevention) are shown in Tables 1 to 3. For acute treatment, recommendations addressed pre-hospital care, emergency care, and acute hospital care. There were more guidelines about ischemic stroke than spontaneous intracerebral hemorrhage (ICH). Strong recommendations were made for reperfusion therapies for acute ischemic stroke. For secondary prevention, strong recommendations included establishing etiological diagnosis, the management of hypertension, weight, diabetes, lipids, and lifestyle modification, and for ischemic stroke, management of atrial fibrillation, valvular heart disease, left ventricular and atrial thrombi, patent foramen ovale, atherosclerotic extracranial and intracranial disease, and antithrombotics in non-cardioembolic stroke. For ICH, strong recommendation was made for application of prothrombin complex concentrate (PCC) with addition of intravenous vitamin K over fresh frozen plasma (FFP) for reversal of vitamin-K-associated ICH, and for external ventricular drainage placement in case of intraventricular hemorrhage (IVH) and hydrocephalus that is contributing to decreased level of consciousness. For rehabilitation, there were strong recommendations for organized stroke unit care, multidisciplinary rehabilitation, task-specific training, fitness training, and specific interventions for post-stroke impairments.

**Table 1.** Acute Management of Stroke—Recommendations, according to minimal, essential, and advance systems of care. See Box 1 for definition of levels of care.

Recommendations	Minimal system	Essential system	Advanced system
A. Early diagnosis and pre-hospital care			
Public education programs about identification of stroke signs and the need to seek emergency care should be designed and implemented in the community	√	√	√
Whenever available, emergency transportation systems should be used to reduce time to ED arrival; emergency dispatch systems should have a method to prioritize possible strokes		√	√
First responders should (1) rapidly evaluate airway, breathing, and circulation to identify and treat a life-threatening situation; (2) use a validated pre-hospital stroke assessment tool; (3) ascertain the time of onset of stroke symptoms (from the patient or witnesses)		√	√
Patients with a possible stroke should be immediately transported to the closest hospital capable of providing emergency stroke care, including IV thrombolysis		√	√
Emergency responders should notify the hospital (pre-hospital notification) that a patient with a possible stroke is en-route to prepare the appropriate hospital resources		√	√
Hospital caring for patients with acute stroke should establish protocols for emergency inter-hospital transfers	√	√	√
Regional systems of stroke care should be developed including two major categories: (1) centers capable of providing initial emergency care, including administration of IV thrombolysis, and (2) centers capable of performing endovascular stroke treatment with comprehensive periprocedural care			√
Stroke centers should (1) have a protocol for emergency stroke evaluation and treatment in the ED; (2) have a designated multidisciplinary stroke team (or access to stroke expertise trough telemedicine); (3) implement a strategy to monitor stroke quality metrics; (4) seek certification by an independent external accreditation entity; (5) ensure continuing stroke education		√	√
**B. Hyperacute hospital care (first hours after stroke)**			
Hospitals caring for patients with acute stroke should have an organized protocol for the emergency evaluation of patients with suspected stroke using a validated stroke screening tool		√	√
Telemedicine/telestroke resources and systems should be supported by health care institutions and governments to ensure availability of stroke expertise coverage 24/7 wherever it is not available on site		√	√
Tracheal intubation is indicated for a compromised airway or insufficient ventilation due to impaired alertness or bulbar dysfunction		√	√
Supplemental oxygen should be provided to maintain oxygen saturation ⩾94%	√	√	√
Hypotension and hypovolemia should be corrected to maintain systemic perfusion levels necessary to support organ function	√	√	√
Emergency treatment of hypertension is indicated if there is concomitant acute myocardial ischemia, aortic dissection, or preeclampsia/eclampsia	√	√	√
Capillary blood glucose should be checked immediately in suspected stroke. Hypoglycemia (glucose below 60 mg/dL or 3.3 mmol/L) should be treated with IV dextrose	√	√	√
Electrocardiography and other blood tests (complete cell count, serum electrolytes and creatinine, INR and partial thromboplastin time, serum troponin) should be obtained, but should not delay the initiation of reperfusion therapy	√	√	√
A stroke severity rating scale (e.g. NIHSS) should be used in the ED		√	√
All patients with suspected acute stroke should undergo brain imaging (head CT or brain MRI) without delay upon hospital arrival and before receiving any specific treatment for stroke		√	√
***Reperfusion therapy for AIS***			
Patients eligible for IV thrombolysis should have the treatment initiated as soon as possible		√	√
IV alteplase (0.9 mg/kg, maximum dose 90 mg over 60 min with initial 10% of dose given as bolus over 1 min) is recommended for selected patients who can be treated within 4.5 h of ischemic stroke symptom onset or last known well. Contraindications for IV thrombolysis are shown in (Supplemental Appendix 4)		√	√
In patients with AIS who awake with stroke symptoms or have unclear time of onset >4.5 h from last known well, IV alteplase administered within 4.5 h of stroke symptom recognition can be beneficial if MRI shows DWI-FLAIR mismatch			√
For patients with AIS within 4.5–9 h of symptom onset who have CT or MRI core/perfusion mismatch, and for whom mechanical thrombectomy is either not indicated or not planned, consider intravenous thrombolysis with alteplase			√
Only the assessment of blood glucose must precede the initiation of IV alteplase in all patients		√	√
Patients with AIS and acute hypertension who are otherwise eligible for IV thrombolysis should have their BP lowered below 185/110 mm Hg before IV thrombolysis is initiated		√	√
Eligible patients should receive IV thrombolysis even if mechanical thrombectomy is being considered. Do NOT evaluate response to IV thrombolysis before proceeding with catheter angiography for mechanical thrombectomy		√	√
Patients with clinically suspected LVO should have non-invasive angiography (e.g. CTA)		√	√
Patients with AIS within 6–24 h of time last known well who have a LVO in the anterior circulation should have advanced imaging (CTP or DW-MRI, with or without MRI perfusion) to determine eligibility for mechanical thrombectomy			√
Patients should receive mechanical thrombectomy with a stent retriever or with direct aspiration if they meet all the following criteria: (1) age ⩾ 18 years; (2) pre-stroke mRS score of 0–1; (3) causative occlusion of the internal carotid artery or MCA (M1); (4) NIHSS score of ⩾ 6; (5) ASPECTS of ⩾ 6; and (6) treatment can be initiated (groin puncture) within 6 h of symptom onset or last known well			√
Mechanical thrombectomy is also recommended between 6 and 24 h in patients who have sizable mismatch between ischemic core (by CTP or MRI-DWI) and either clinical deficits or area of hypoperfusion (by CTP or MRI-PWI)			√
Mechanical thrombectomy can be considered in patients with an occlusion or stenosis of the cervical ICA in addition to an intracranial LVO			√
The technical goal of mechanical thrombectomy should be reperfusion to a modified Thrombolysis in Cerebral Infarction (mTICI) grade 2b/3			√
***Intracerebral hemorrhage***			
In patients with lobar spontaneous ICH and age < 70 years, deep/posterior fossa spontaneous ICH and age < 45 years, or deep/ posterior fossa and age 45–70 years without history of hypertension, head CTA plus consideration of venography is recommended to exclude macrovascular causes or cerebral venous thrombosis		√	√
In patients with spontaneous IVH and no detectable parenchymal hemorrhage, catheter angiography is recommended to exclude a vascular anomaly			√
In patients with spontaneous ICH and non-invasive angiography suggestive of a vascular anomaly, catheter angiography is recommended to confirm and, if possible, treat the vascular anomaly			√
In case of IVH and hydrocephalus that is contributing to decreased level of consciousness, external ventricular drainage is recommended			√
In patients with spontaneous ICH and hypertension presenting within 6 h of symptom onset, we recommend acute lowering of SBP to a target of 140 mm Hg (strictly avoiding SBP < 110 mm Hg) to reduce the risk of hematoma expansion	√	√	√
In patients with anticoagulant-associated spontaneous ICH, anticoagulation should be discontinued immediately, and anticoagulation should be reversed as soon as possible	√	√	√
In patients with VKA-associated spontaneous ICH and INR ⩾ 2.0, 4-factor (4F) prothrombin complex concentrate (PCC) is recommended over fresh-frozen plasma (FFP). FFP or 3F-PCC should be used when 4F-PCC is not available. IV vitamin K should be administered shortly after 4F-PCC or FFP to prevent later re-emergence of anticoagulation		√	√
For heparin-related ICH, protamine sulfate is recommended		√	√
In patients with ICH who were taking a direct oral anticoagulant, idarucizumab is recommended for reversal of dabigatran and andexanet alpha or, if not available, 4F-PCC for reversal of factor Xa inhibitors			√
***Cerebral venous sinus thrombosis***			
A non-invasive venogram (CTV or MRV) should be performed in suspected CVST if the plain CT or MRI are inconclusive		√	√
In patients with suspected or confirmed CVST, possible causative infections should be excluded	√	√	√
Anticoagulation should be started immediately after the diagnosis of CVST, even if intracranial hemorrhage is present. IV heparin or subcutaneous LMWH can be used	√	√	√
**C. Acute In-Hospital care (first days after stroke)-medical management (for organization of care, dysphagia, mobilization, skin care, and continence, see rehabilitation Table 3)**			
Patients should be admitted to a stroke unit (a specialized, geographically defined hospital unit dedicated to the management of stroke patients) or, if critically ill, to an intensive care unit (see also rehabilitation recommendations)		√	√
Cardiac monitoring is recommended to screen for atrial fibrillation and other potentially serious cardiac arrhythmias for at least the first 24 h		√	√
In patients with AIS, blood pressure should be maintained below 180/105 mm Hg for at least the first 24 h after acute reperfusion treatment		√	√
In patients with AIS, administration of aspirin is recommended within 24–48 h after stroke onset. For those treated with IV thrombolysis, aspirin administration is generally delayed until > 24 h. Patients with aspirin allergy should be given an alternative antiplatelet medication	√	√	√
Gradual early mobilization should be encouraged. Patients who have limited mobility should be treated with thigh-high intermittent pneumatic compression devices (IPC), if available	√	√	√
Body temperature should be monitored and fever (temperature > 38°C) should be treated. Sources of fever should be investigated and treated	√	√	√
Antiseizure medications are only indicated for documented secondary seizures	√	√	√
***Massive Stroke***			
The management of patients with massive stroke should be reached by shared decision making with participation of patient (when possible) and family taking into consideration the anticipated prognosis for functional recovery	√	√	√
Patients with massive cerebral or cerebellar infarction or hemorrhage or at risk of malignant swelling should be rapidly transferred to a center with neurosurgical expertise if their condition is deemed survivable	√	√	√
In patients with massive strokes, serial physical examinations (and repeat head CT scan when appropriate) should be performed to identify worsening brain swelling		√	√
Patients with massive strokes should be immediately intubated if they develop neurological deterioration with respiratory insufficiency		√	√
Decompressive hemicraniectomy is indicated within 48 h of symptom onset in patients with massive hemispheric infarction and worsening neurological condition (functional benefit much greater in patients < 60 years)			√
Ventriculostomy is recommended in the treatment of symptomatic obstructive hydrocephalus after cerebellar infarction. Concomitant or subsequent decompressive suboccipital craniectomy is indicated if brainstem compression is present			√
Patients with spontaneous ICH (with or without IVH) and symptomatic hydrocephalus should be treated with ventricular drainage			√
Patients with cerebellar ICH who develop neurological deterioration, have brainstem compression, and/or have hydrocephalus from ventricular obstruction should be treated with decompressive suboccipital craniectomy (with or without ventricular drainage)			√

ED: emergency department; IV: intravenous; INR: international normalized ratio; NIHSS: National Institute of Health Stroke Scale; MRI: magnetic resonance imaging; AIS: acute ischemic stroke; DWI: diffusion weighted imaging; BP: blood pressure; LVO: large vessel occlusion; CTA: computed tomography angiography; CTP: computed tomography perfusion, MCA: middle cerebral artery;, ICH: intracerebral hemorrhage; IVH: intraventricular hemorrhage; SBP: systolic blood pressure; VKA, PCC: prothrombin complex concentrate; FFP: Fresh frozen plasma, CTV: cerebral venogram, MRV: magnetic resonance venogram; CVST: cerebral venous sinus thrombosis; LMWH: low molecular weight heparin; IPC: intermittent pneumatic compression; AIS: acute ischemic stroke; ASPECTS: Alberta Stroke Program Early CT Score; FLAIR: Fluid-attenuated inversion recovery; PWI: perfusion-weighted imaging; ICA: internal carotid artery; VKA: Vitamin K antagonist.

**Table 2.** Secondary Prevention after Stroke—Recommendations.

Recommendations	Minimal	Essential	Advanced
**Diagnosis**			
**Etiological diagnosis**			
A diagnostic evaluation of stroke etiology should be started or ideally completed within 48 h of stroke onset		√	√
**Blood work**			
Blood work including complete blood count, prothrombin time, partial thromboplastin time, glucose, HbA1c, creatinine, and fasting or non-fasting lipid profile, is recommended in patients with ischemic stroke or TIA	√	√	√
**Cerebral imaging**			
In patients with a suspicion of an ischemic cerebrovascular event (stroke or TIA), neuroimaging with CT or MRI is recommended		√	√
**Vascular imaging**			
Vascular imaging of the extracranial cervical arteries is recommended to identity patients with severe internal carotid artery stenosis who may benefit from urgent carotid endarterectomy or stenting. Imaging can be performed with carotid Doppler ultrasound, CTA, or MRA		√	√
**Electrocardiographic monitoring**			
Patients with an ischemic stroke or TIA should have an ECG to screen for atrial fibrillation	√	√	√
Patients with an ischemic stroke or TIA should have at least 24 h of cardiac monitoring to screen for atrial fibrillation			√
Patients with an embolic ischemic stroke or TIA without atrial fibrillation on initial short-term ECG should have longer-term monitoring, for at least 14 days to screen for atrial fibrillation			√
**Hypertension**			
**Need for blood pressure management**			
Blood pressure should be assessed and treated in all patients with ischemic and hemorrhagic stroke and TIA	√	√	√
**Timing of blood pressure treatment**			
Blood pressure treatment should be initiated as soon as possible after a stroke or TIA, or at least before discharge	√	√	√
**Selection of antihypertensive drugs**			
Angiotensin-converting enzyme-inhibitors combined with a thiazide diuretic can reduce the risk of stroke in ischemic stroke or TIA patients with and without a diagnosis of hypertension and are therefore favored in patients with ischemic stroke or TIA	√	√	√
In patients with ischemic stroke or TIA and hypertension, any of the following antihypertensive drugs can be used: thiazide diuretic, angiotensin-converting enzyme inhibitor, or angiotensin II receptor. Beta-blockers may be used in patients with ischemic heart disease	√	√	√
In patients with ischemic stroke or TIA and hypertension, an individualized approach to the selection of antihypertensive medications based on comorbidities is recommended	√	√	√
**Blood pressure targets**			
The target blood pressure for patients with stroke or TIA is < 130/80 mm Hg	√	√	√
**Weight**			
In patients with ischemic stroke or TIA, it is recommended to estimate the BMI at the time of the event and during long-term follow-up	√	√	√
In patients with ischemic stroke or TIA who are overweight or obese, weight loss is recommended	√	√	√
In patients with ischemic stroke or TIA who are obese, referral to a multidisciplinary lifestyle modification program is recommended			√
**Diabetes**			
In patients with ischemic stroke or TIA and diabetes, a target of HbA1c ⩽ 7% is recommended	√	√	√
In patients with ischemic stroke or TIA and diabetes, the treatment of diabetes should include glucose-lowering agents with demonstrated efficacy for reducing vascular outcomes	√	√	√
In patients with ischemic stroke or TIA and diabetes, a transdisciplinary team approach is recommended			√
**Lipids**			
Ischemic stroke and TIA patients without a proven cardioembolic mechanism and an LDL-cholesterol level > 2.5 mmol/L (> 100 mg/dL) should receive atorvastatin 80 mg to reduce stroke recurrence	√	√	√
The target LDL-cholesterol level in patients with ischemic stroke and TIA should be < 1.8 mmol/L (70 mg/dL)	√	√	√
The target LDL-cholesterol level in patients with ischemic stroke and TIA and atherosclerotic disease of the extracranial or intracranial arteries should be 1.8 mmol/L (70 mg/dL). Ezetimibe can be added to Atorvastatin to reach this goal	√	√	√
In patients with ischemic stroke or TIA in whom a target LDL-cholesterol level is not achievable, a consideration should be made to refer to an expert in lipid management for adding a PCSK9 inhibitor			√
In patients with ischemic stroke or TIA on lipid-lowering medications, lipid levels should be monitored 1–3 months after treatment initiation, followed by regular assessments and dose adjustments every 3–12 months thereafter	√	√	√
**Lifestyle modification**			
**Physical activity**			
In patients with ischemic stroke or TIA who can engage in physical activity, low/moderate-intensity aerobic activity for 10 min 4 days/week, or vigorous aerobic activity for 20 min twice a week is recommended	√	√	√
**Smoking cessation**			
In patients with ischemic stroke or TIA, smoking status should be evaluated and documented in all health care encounters	√	√	√
In patients with ischemic stroke or TIA who are active smokers, counseling with or without pharmacological therapy with nicotine replacement, bupropion, or varenicline is recommended	√	√	√
In patients with ischemic stroke or TIA who are active smokers, a referral to a smoking cessation clinic is recommended if available			√
**Alcohol consumption**			
In patients with ischemic stroke or TIA who drink > 2 alcoholic drinks daily for men or > 1 alcoholic drink daily for women, counseling for alcohol intake reduction is recommended. Specialized services are also recommended	√	√	√
In patients with ischemic stroke or TIA with alcohol used disorder, specialized services are recommended			√
**Recreational drugs**			
In patients with ischemic stroke or TIA who use stimulant recreational drugs, counseling is recommended	√	√	√
In patients with ischemic stroke or TIA who use recreational drugs, specialized services are recommended			√
**Salt consumption**			
In patients with ischemic stroke or TIA, salt intake should be reduced, at least < 2000 mg daily	√	√	√
**Atrial fibrillation**			
**Oral anticoagulation**			
In patients with ischemic stroke or TIA and valvular (mechanical valve replacement or moderate/severe mitral stenosis) atrial fibrillation, oral anticoagulation is recommended	√	√	√
In patients with ischemic stroke or TIA with nonvalvular atrial fibrillation or flutter, either paroxysmal, persistent, or permanent, oral anticoagulation is recommended	√	√	√
In patients with ischemic stroke or TIA with nonvalvular atrial fibrillation, direct oral anticoagulants are preferred over vitamin-K antagonists	√	√	√
In patients with ischemic stroke or TIA with nonvalvular atrial fibrillation, who are receiving vitamin-K antagonists and cannot achieve a consistent INR level, direct oral anticoagulants are preferred over vitamin-K antagonists		√	√
**Antiplatelet therapy**			
Patients who are suitable for anticoagulation should not receive antiplatelets for secondary stroke prevention	√	√	√
**Valvular heart disease**			
In patients with a mechanical mitral valve and a previous ischemic stroke or TIA before the valve replacement, add aspirin 75–100 mg daily to warfarin targeting an INR range of 2.5–3.5	√	√	√
In patients with ischemic stroke or TIA who have native aortic or nonrheumatic mitral valve disease and no AF or any other indication for anticoagulation, antiplatelet agents are recommended	√	√	√
In patients with ischemic stroke or TIA who have a bioprosthetic aortic or mitral valve replacement without any other indication for anticoagulation, long-term antiplatelet therapy is recommended beyond the 3- to 6-month window after the procedure	√	√	√
**Left ventricular and atrial thrombus**			
In patients with ischemic stroke or TIA and a left ventricular thrombus, anticoagulation at least for 3 months is recommended	√	√	√
In patients with ischemic stroke or TIA and left atrial or left atrial appendage thrombus in the context of ischemic, nonischemic, or restrictive cardiomyopathy and LV dysfunction, oral anticoagulation with vitamin-K antagonists is recommended for at least 3 months	√	√	√
**Patent foramen ovale**			
**Neurocardiology assessment**			
In patients with a cryptogenic stroke and a PFO, a team-based approach by a cardiologist and a neurologist is recommended to identify the causal role of the PFO and to define the best therapeutic approach			√
**PFO closure**			
Patients with non-lacunar ischemic stroke and a PFO should undergo PFO closure if they are aged between 18 and 60 years, if PFO is considered causal			√
**Atherosclerotic extracranial large vessel disease**			
**Urgent evaluation**			
Patients with an acute ischemic stroke or TIA and ipsilateral internal carotid artery stenosis of 50–99% should be evaluated urgently by an expert team to decide carotid revascularization			√
**Carotid endarterectomy**			
Patients with an acute ischemic stroke or TIA in the past 6 months and ipsilateral extracranial internal carotid artery stenosis of 70–99% should be offered a carotid endarterectomy if the morbidity/mortality risk of the surgical team is < 6%			√
Patients with an acute ischemic stroke or TIA in the past 6 months and ipsilateral extracranial internal carotid artery stenosis of 50–69% could be offered a carotid endarterectomy depending on individual characteristics, including age, sex, and comorbidities if the morbidity/mortality risk of the surgical team is < 6%. The benefit of carotid endarterectomy in patients with 50–69% stenosis is substantially lower than in those with ⩾ 70%. In patients with 50–69% stenosis, carotid endarterectomy is associated with a higher risk of poor outcomes in the first 2 post-procedural years. However, there is a significant benefit from surgery for any stroke or operative death at 5 years.^203^ As such, an estimated life expectancy > 5 years is required in this group. In addition, there was no clear benefit of carotid endarterectomy in the NASCET trial for women with 50–69% stenosis^204^			√
Carotid endarterectomy if indicated, should be performed as early as possible if the patient is clinically stable, ideally within 14 days after symptoms onset			√
Carotid endarterectomy is preferred over carotid stenting			√
**Carotid stenting**			
Carotid stenting may be considered for patients who are NOT candidates for a carotid endarterectomy (e.g. technical, anatomic, or medical reasons)			√
**Medical therapy**			
In patients with ischemic stroke or TIA and carotid or vertebral artery stenosis, intensive medical therapy (e.g. antiplatelet agents, lipid-lowering medications, blood pressure management, and diabetes control) is recommended, regardless of whether a revascularization procedure is done, in addition to diet, exercise, and smoking cessation	√	√	√
In patients with ischemic stroke or TIA and aortic arch atheroma, antiplatelet therapy is recommended	√	√	√
In patients with ischemic stroke or TIA and aortic arch atheroma, a target LDL-cholesterol of 1.8 mmol/L (70 mg/dL) should be pursued with high-dose statin therapy	√	√	√
**Intracranial atherosclerotic disease**			
**Anticoagulation**			
In patients with acute ischemic stroke or TIA due to high-grade intracranial atherosclerotic disease, the use of anticoagulants is not recommended unless there is another indication for anticoagulation (e.g. atrial fibrillation)	√	√	√
**Single antiplatelet therapy**			
In patients with ischemic stroke or TIA caused by moderate to high-grade intracranial atherosclerotic stenosis (50–99%), aspirin 325 mg daily is recommended over oral anticoagulation. There are no strong recommendations supporting the use of dual antiplatelet therapy (DAPT) over single antiplatelet therapy (SAPT) in this population. While the Stenting vs Aggressive Medical Therapy for Intracranial Arterial Stenosis (SAMMPRIS) trial showed that DAPT is better than stenting, it did not prove that DAPT is better than SAPT.^205^ A post hoc analysis of the Clopidogrel in High-Risk Patients with Acute Non-disabling Cerebrovascular Events (CHANCE) trial showed no differences in the beneficial effect of DAPT vs SAPT in minor stroke patients with vs without intracranial atherosclerotic disease (ICAD).^206^ In the Clopidogrel Plus Aspirin Versus Aspirin Alone for Reducing Embolization in Patients With Acute Symptomatic Cerebral or Carotid Artery Stenosis (CLAIR) Trial, 93 of 100 patients had symptomatic ICAD. DAPT use was associated with a 54.4% (16.4–75.1) relative risk reduction on microembolic signals on transcranial Doppler ultrasound.^207^	√	√	√
**Blood pressure management**			
In patients with ischemic stroke or TIA caused by moderate to high-grade intracranial atherosclerotic stenosis (50–99%), a systolic blood pressure target of < 140 mm Hg is recommended	√	√	√
**Lipid-lowering agents**			
In patients with ischemic stroke or TIA caused by moderate to high-grade intracranial atherosclerotic stenosis (50–99%), high-dose statin therapy is recommended	√	√	√
**Physical activity**			
In patients with ischemic stroke or TIA caused by moderate to high-grade intracranial atherosclerotic stenosis (50–99%), at least moderate physical activity is recommended	√	√	√
**Angioplasty and stenting**			
In patients with ischemic stroke or TIA and moderate to high-grade intracranial atherosclerotic stenosis (50–99%), angioplasty and stenting is not recommended. Dual antiplatelets is an appropriate medical therapy	√	√	√
**Antithrombotic management in non-cardioembolic stroke**			
**Indication for antiplatelet therapy**			
In patients with non-cardioembolic ischemic stroke or TIA who do not require anticoagulation, long-term antiplatelet therapy is indicated		√	√
**Selection of single antiplatelet agents**			
Antiplatelet agents are recommended for secondary stroke prevention in patients with non-cardioembolic ischemic events who do not require oral anticoagulation, including aspirin 81–325 mg daily, clopidogrel 75 mg daily, or aspirin + dipyridamole 25/200 mg daily	√	√	√
In patients with an acute ischemic stroke or TIA who were not on an antiplatelet agent, a single loading dose of 160 mg should be administered after an intracranial hemorrhage is ruled out on neuroimaging studies		√	√
In patients with an acute ischemic stroke or TIA in whom swallowing mechanisms are impaired, rectal aspirin 325 mg daily, or aspirin 81 mg daily or clopidogrel 75 mg daily administered via enteral tube are reasonable alternatives to oral intake		√	√
**Minor ischemic stroke or TIA**			
In patients with a minor ischemic stroke (NIHSS ⩽ 3) or high-risk TIA (ABCD2 ⩾ 4), DAPT with aspirin 81 mg daily and clopidogrel 75 mg daily should be initiated as early as possible, ideally within 12–24 h of symptoms onset, after an intracranial hemorrhage is excluded on neuroimaging studies. A single loading dose of aspirin (160–325 mg) and clopidogrel (300 mg as per the CHANCE trial or 600 mg as per the POINT trial) should be used at the beginning of DAPT therapy. DAPT is indicated for 21 days and should be followed by long-term single antiplatelet therapy with aspirin 81 mg daily or clopidogrel 75 mg daily		√	√
In patients with mild-moderate ischemic stroke (NIHSS ⩽ 5) or high-risk TIA (ABCD2 ⩾ 4) DAPT with aspirin 75–100 mg daily and ticagrelor 90 mg twice daily should be initiated as early as possible, ideally within 24 h of symptoms onset, after an intracranial hemorrhage is excluded on neuroimaging studies. A single loading dose of aspirin (300–325 mg) and ticagrelor (180 mg) should be used at the beginning of DAPT therapy. DAPT is indicated for 30 days and should be followed by long-term single antiplatelet therapy		√	√
**Embolic stroke of undetermined source (ESUS)**			
Patients with ESUS should not receive oral anticoagulants. The recommended antithrombotic regimen for secondary stroke prevention in ESUS patients is antiplatelet therapy		√	√
**Extracranial artery dissection**			
In patients with ischemic stroke or TIA and extracranial carotid or vertebral artery dissection, either antiplatelet therapy or oral anticoagulants are recommended for at least 3 months		√	√
**Carotid web**			
In patients with ischemic stroke or TIA and a carotid web in the ipsilateral vascular territory, antiplatelet therapy is recommended		√	√
**Fibromuscular dysplasia**			
In patients with ischemic stroke or TIA and fibromuscular dysplasia, antiplatelet therapy and lifestyle modification are recommended		√	√
**Positive anti-phospholipid antibodies**			
In patients with ischemic stroke or TIA and positive anti-phospholipid who do not fulfill criteria for anti-phospholipid syndrome, antiplatelet therapy is recommended. There are no strong recommendations for patients with confirmed anti-phospholipid syndrome regarding antithrombotic therapy		√	√
**Sickle cell disease**			
In patients with ischemic stroke or TIA and sickle cell disease, chronic blood transfusions are recommended to reduce hemoglobin S to < 30% of total hemoglobin		√	√
**Vasculitis**			
In patients with ischemic stroke or TIA and symptoms of giant cell arteritis, immediate treatment with high-dose steroids should be initiated	√	√	√
In patients with ischemic stroke or TIA and infectious vasculitis (VZV, bacterial, or other infectious agents), the underlying infection should be treated	√	√	√
**Spontaneous intracerebral hemorrhage**			
Aggressive long-term blood pressure monitoring, treatment, and control are recommended	√	√	√
**Cerebral venous sinus thrombosis**			
Anticoagulation with warfarin (or dabigatran) is recommended for a minimum of 3–6 months	√	√	√

ABCD2: age, blood pressure, clinical, duration, diabetes; TIA: transient ischemic attack; CT: computed tomography; MRI: magnetic resonance imaging; CTA: computed tomography angiography; ECG: electrocardiogram; BMI: body mass index; LDL: low-density lipoprotein; INR: international normalized ratio; PFO: patent foramen ovale; NIHSS: National Institute of Health Stroke Scale; MRA: magnetic resonance angiography; AF: atrial fibrillation.

**Table 3.** Rehabilitation after Stroke—Recommendations.

Recommendation	Minimal	Essential	Advanced
**Organization of hospital care and principles of rehabilitation**			
Acute stroke patients admitted to hospital should have an initial assessment by rehabilitation professionals as soon as possible after admission		√	√
Stroke patients should be treated on a specialized, geographically defined stroke rehabilitation unit, with coordinated care, staffed by an interdisciplinary rehabilitation team (physicians, nurses, physiotherapists, occupational therapists, speech-language therapists, and social workers and dieticians) with expertise/training in stroke rehabilitation, recovery, and return to work		√	√
Patient, family, and caregiver education should be provided formally and informally	√	√	√
Person-centered, collaborative, goal setting with patients and their families is recommended, clearly communicated and documented, regularly reviewed, including around transitions of care	√	√	√
Rehabilitation should include as much scheduled task-specific therapy as possible, to meet optimal recovery and tolerability		√	√
Commence mobilization within 48 h of stroke onset unless otherwise contraindicated but do not start intensive out-of-bed activities within 24 h of stroke onset		√	√
Stroke patients and their families/carers should have access to specialist palliative care teams and care consistent with the principles and philosophies of palliative care		√	√
**Skin care**			
Regular skin assessments are recommended with objective scales of risk, e.g. Braden scale	√	√	√
Minimize skin friction and skin pressure, provide appropriate support surfaces, avoid excessive moisture, maintain adequate nutrition and hydration to prevent skin breakdown	√	√	√
Regular turning, good skin hygiene, and use of specialized mattresses, wheelchair cushions, and seating are recommended until mobility returns	√	√	√
**Management of dysphagia and provision of food and fluids**			
Health care professionals should regularly monitor and reassess patients with dysphagia who need modified food and liquid	√	√	√
Ensure good oral and dental (including dentures) hygiene, particularly for those with dysphagia, through assistance and/or education	√	√	√
For stroke survivors with swallowing difficulties, behavioral approaches such as swallowing exercises (e.g. shaker exercises, or chin tuck against resistance), environmental modifications, safe swallowing advice, and appropriate dietary modifications should be used early	√	√	√
Offer swallowing therapy, e.g. compensatory strategies, exercises and postural advice, at least 3 times a week to people with dysphagia after stroke who are able to participate, for as long as they make functional gains.		√	√
For patients whose nutrition status is poor or deteriorating, nutrition supplementation should be offered. Enteral diet should be started if required within 7 days of admission (preferably within 24–48 h)		√	√
Patients who require tube feeding should receive percutaneous endoscopic gastrostomy rather than nasogastric tube in the post-acute phase		√	√
**Aerobic training**			
Individually tailored aerobic training involving large muscle groups (with monitoring of heart rate and blood pressure) should be incorporated into a comprehensive stroke rehabilitation program to enhance cardiovascular endurance and cognitive function	√	√	√
Exercise is needed at least 3 times weekly for a minimum of 8 weeks, progressing as tolerated to 20 min or more per session, exclusive of warm-up and cool-down	√	√	√
Use strategies to address specific barriers to physical activity related to patients, health care providers, family, and/or the environment	√	√	√
Group circuit class therapy should be used to increase scheduled therapy time		√	√
Offer early supported discharge services for those with mild to moderate disability		√	√
Provide advice on prescribed medications for people after stroke	√	√	√
Support and educate people after stroke and their families and carers, in relation to emotional adjustment to stroke, recognizing that psychological needs may change over time and in different settings	√	√	√
**Specific impairments: upper and lower limb**			
Stroke survivors with difficulty walking should undertake tailored repetitive practice of walking (or components of walking) using circuit class therapy (with a focus on overground walking practice and/or Treadmill training with or without body weight support)		√	√
Rhythmic auditory stimulation (RAS) could be considered for improving gait parameters, including gait velocity, cadence, stride length, and gait symmetry			√
For reduced strength in arms or legs, provide progressive resistance training which is meaningful, engaging, repetitive, progressively adapted, task-specific, and goal-oriented		√	√
Non-invasive brain stimulation, including repetitive transcranial magnetic stimulation (rTMS) could be considered as an adjunct to upper extremity therapy			√
For stroke survivors with some active wrist and finger extension, traditional or modified intensive constraint-induced movement therapy should be provided		√	√
For stroke survivors who have difficulty sitting, practising reaching beyond arm’s length while sitting with supervision/ assistance should be undertaken	√	√	√
For stroke survivors who have difficulty in standing up from a chair, practice of standing up should be undertaken	√	√	√
For stroke survivors who have difficulty with standing, activities that challenge balance should be provided	√	√	√
Force platform biofeedback should be used for people with difficulty standing		√	√
Consider mirror therapy for patients with very severe paresis		√	√
Functional Electrical Stimulation (FES) of the wrist and forearm muscles should be considered to reduce motor impairment and improve function and for people with gait disturbance			√
Virtual reality, including both immersive technologies such as head mounted or robotic interfaces and non-immersive technologies such as gaming devices can be used as adjunct tools to other rehabilitation therapies, to provide additional opportunities for engagement, feedback, repetition, intensity, and task-oriented training			√
Chemo-denervation using botulinum toxin can be used to increase range of motion and decrease pain for patients with focal symptomatically distressing spasticity (upper and lower limbs)			√
Ankle-foot orthoses should be used on selected patients with foot drop following proper assessment and with follow-up to verify effectiveness	√	√	√
Mental Practice should be considered for upper and low limb motor re-training		√	√
**Vision**			
Screen for visual problems		√	√
For visual neglect, use interventions focused on functional tasks		√	√
Refer people with double vision for formal orthoptic assessment		√	√
**Aphasia**			
Provide speech and language therapy, within 4 weeks post-stroke, to improve functional communication, communication aids, reading comprehension, general expressive language, written language and supported conversation techniques for potential communication partners		√	√
Adapt all written material for people with aphasia		√	√
Help and enable people with communication problems to communicate everyday needs and wishes and to support decision making	√	√	√
**Prevention and treatment of shoulder subluxation**			
Health care staff, patients, and family should be educated to correctly protect, position, and handle the involved arm	√	√	√
Taping of the affected shoulder can reduce pain	√	√	√
**Mood disorders**			
Patients with acute stroke should be screened for depression using a structured depression assessment tool	√	√	√
Treatment for post-stroke depression may include psychotherapy and/or antidepressants		√	√
For severe, persistent, or troublesome tearfulness, emotional incontinence or lability, consider a trial of antidepressants		√	√
**Assessment and management of cognitive functions**			
Treat hypertension to reduce the risk of cognitive decline	√	√	√
Use interventions for visual neglect after stroke that focus on the relevant functional tasks		√	√
**Continence**			
For urinary incontinence, identify and manage its cause (e.g. infection, constipation, urinary retention, overactive bladder)	√	√	√
For fecal incontinence, identify and manage its cause (e.g. infection, fecal impaction, and overflow)	√	√	√
Prolonged use of indwelling bladder catheters should be avoided as much as possible to reduce risk of urinary tract infection	√	√	√
A bowel management program should be implemented for persistent constipation or bowel incontinence	√	√	√
**Neuropathic pain**			
Offer a choice of amitriptyline, duloxetine, gabapentin, or pregabalin as initial treatment for neuropathic pain; if the initial treatment is not effective or is not tolerated, offer one of the remaining three drugs, and consider switching again if the second and third drugs tried are also not effective or not tolerated. Consider capsaicin cream for people with localized neuropathic pain who wish to avoid, or who cannot tolerate, oral treatments		√	√
**Discharge from hospital and ongoing care**			
Before discharge, agree a health and social care plan with the patient and family/carer; establish they have a safe and enabling home environment, check carers have the support they need	√	√	√
People with disabilities after stroke should be followed up within 72 h by the specialist stroke rehabilitation teams		√	√
People with ongoing rehabilitation goals and those with difficulty performing activities of daily living should have ongoing access to specialized stroke services after leaving hospital		√	√
A planned transition from structured aerobic exercise (in hospital) to more self-directed physical activity at home or in the community should be implemented	√	√	√
Encourage participation in evidence-based community exercise programs		√	√
Review health and social care needs of people after stroke and their caregivers at 6 months and annually thereafter. This includes people in care homes		√	√
Hospitals should maintain a data collection system to monitor performance metrics. Participation in an external stroke data repository is also recommended		√	√

The performance metrics derivable from these recommendations are shown in Table 4.

**Table 4.** **Performance metrics.** Appropriate denominator and exclusions need to be established and applied.

Performance metrics
**Acute Management**
Time from stroke onset to assessment by health care professional (in min/h)
Proportion of stroke and TIA evaluated with stroke severity rating scale
Proportion of stroke and TIA patients receiving a CT scan within 25 min of hospital arrival
Door-to-needle time for ischemic stroke patients who receive IV thrombolysis
Proportion of ischemic stroke patients who are treated with IV thrombolysis
Proportion of eligible ischemic stroke patients who receive mechanical thrombectomy
Proportion of eligible ischemic stroke patients who receive mechanical thrombectomy in the late time window (>6 h)
Proportion of (mTICI) grade 2b/3 after mechanical thrombectomy
Time from hospital arrival to start of antihypertensive therapy in patients with ICH and hypertension
Proportion of patients with ICH achieving adequate control of hypertension in the first 24 h
Time from hospital arrival to administration of adequate anticoagulation reversal treatment in patients with anticoagulation-related ICH
Proportion of patients with CVST who are started on therapeutic anticoagulation within 24 h of the diagnosis
Proportion of stroke patients who are admitted to an acute stroke unit
Proportion of stroke patients who are screened or assessed for swallowing deficits
Proportion of patients with ischemic stroke who receive aspirin within the first 48 h
Proportion of stroke patients who receive DVT prophylaxis
Time from stroke onset until first mobilization
Proportion of eligible patients with large hemispheric infarction who are treated with hemicraniectomy
Proportion of stroke patients who receive education about stroke
Distribution of discharge locations for stroke patients
**Secondary Prevention**
Proportion of stroke or TIA patients with diagnostic evaluation of stroke etiology started within 48 h of stroke onset
Proportion of stroke or TIA patients with complete brain and extracranial/intracranial vascular imaging
Proportion of stroke or TIA patients with at least 24 h of cardiac monitoring
Proportion of stroke or TIA patients with their blood pressure assessed and treated
Proportion of stroke or TIA patients with their BMI estimated at the time of the event
Proportion of stroke or TIA patients with diabetes who achieve an HbA1c ⩽ 7%
Proportion of stroke or TIA patients with who achieve an LDL-cholesterol < 1.8 mmol/L or 70 mg/dL
Proportion of stroke or TIA patients who engage in low/moderate-intensity aerobic activity for 10 min 4 days/week
Proportion of stroke or TIA patients who are active smokers that receive smoking cessation counseling
Proportion of stroke or TIA patients who drink > 2 alcoholic drinks/d daily for men or > 1 alcoholic that receive counseling for alcohol intake reduction
Proportion of stroke or TIA patients with salt intake < 2000 mg daily
Proportion of stroke or TIA patients with atrial fibrillation who receive oral anticoagulants
Proportion of cryptogenic stroke or TIA patients with a PFO who are assessed by a Neurocardiology team or a cardiologist and a neurologist
Proportion of cryptogenic stroke patients with PFO, aged 18–60 years, who receive a PFO closure
Proportion of stroke or TIA patients with severe ipsilateral internal carotid artery stenosis who receive an endarterectomy within 14 days after symptoms onset
Proportion of minor stroke (NIHSS ⩽ 3 for clopidogrel or NIHSS ⩽ 5 for ticagrelor) or high-risk TIA (ABCD2 ⩾ 4) patients who receive dual antiplatelet therapy within 24 h of symptoms onset
Proportion of stroke or TIA patients with no indication for anticoagulation or dual antiplatelet therapy who receive a single antiplatelet agent for secondary stroke prevention
**Rehabilitation**
Proportion of patients assessed by a multidisciplinary team of stroke specialists
Proportion of patients/family receiving education about stroke
Proportion of patients/family receiving person centered, collaborative goal setting
Amount of task-specific therapy (minutes per day)
Proportion commencing mobilization between 24 and 48 h
Proportion of patients predicted to die who have access to specialist palliative care teams
Proportion of patients (especially those who are immobile) who have regular assessments of skin care and then measures taken to reduce the risk of skin breakdown
For patients unable to meet nutritional requirements, proportion receiving nutritional support (oral or with tube feeding), and interval until its institution
Proportion of patients having aerobic training at least three times weekly
Proportion of people with mild to moderate disability receiving early supported discharge
Proportion of people who receive advice on medication
Proportion of patients who receive emotional support
Proportion of patients who receive repetitive task training for their respective impairments (see individual impairments)
Proportion of patients with focal symptomatic distressing spasticity (upper and lower limbs) who receive chemo-denervation using botulinum toxin
Proportion of patients with foot drop getting an ankle-foot orthoses
Proportion of people with double vision referred for formal orthoptic assessment
Proportion of patients with aphasia evaluated by speech and language therapy
Proportion of all patients receiving appropriate handling to avoid shoulder subluxation including correct position of the hemiparetic arm and avoidance of overhead pulleys
Proportion of patients with acute stroke who are screened for depression using a structured depression assessment tool
For patients with a diagnosis of depression, proportion receiving psychotherapy and/or antidepressants
Proportion of patients who have their cognitive function assessed
Proportion of patients who have access to force platform biofeedback and mirror therapy
Proportion of patients receiving, and being discharged with, indwelling bladder catheters (lower proportion is better)
For people with constipation or bowel incontinence, the proportion with a bowel management program
Proportion of people with ongoing rehabilitation goals and those with difficulty performing activities of daily living who have access to specialized stroke services after leaving hospital
Proportion participating long-term in community-based exercise programs
Proportion of patients receiving a 6-month review
Proportion of hospitals within a region which maintains a data collection system to monitor performance metrics

ABCD2: age, blood pressure, clinical, duration, diabetes; TIA: transient ischemic attack; CT: computed tomography; IV: intravenous; ICH: intracerebral hemorrhage; CVST: cerebral venous sinus thrombosis; BMI: body mass index; LDL: low-density lipoprotein; PFO: patent foramen ovale; NIHSS: National Institute of Health Stroke Scale; DVT: Deep venous thrombosis.

### Stakeholder assessment

We received several responses from health care professional members of the WSO board in November 2022, which resulted in an updated version of the manuscript. This updated version was further reviewed by PWLE. The latter assessment determined that this document was thorough enough to address their most significant concerns. It also identified topics that were not covered in the present work because of the lack of strong recommendations in currently available guidelines (e.g. left atrial appendage for stroke prevention in patients with high-bleeding risk). PWLE also mentioned their concern about guidelines not consistently applying the same criteria for assessing the strength of recommendations, possibly resulting in disparities in stroke care across countries/regions (e.g. measuring troponin levels in the acute stroke setting). They recommended patients should be involved in the preparation of guidelines from the beginning, and guidelines should include sections with lay language for patients to understand what they mean. Clear timelines are very important for acute treatment, secondary prevention, and rehabilitation. Overall, they felt satisfied with the systematic approach and contents of the reviewed document. Comments from one more PWLE received in January 2023 were the need for single rooms in the acute phase, online support groups, and re-training for stroke survivors unable to return to their previous jobs.

## Discussion

### Main findings of our searches

To the best of our knowledge, this is the first systematic review and synthesis of all available stroke guidelines worldwide, published from 1 January 2011. The scope varied from specific issues to all aspects of the stroke pathway. The full list of publications we initially identified is displayed in our reference list^3–202^ and those excluded (e.g. because they superseded) are shown in Supplemental Appendix 3.

### Main recommendations for acute stroke

Most guidelines for acute management of acute ischemic stroke were focused on acute reperfusion therapies, though some provided a more comprehensive discussion and broader management guidance. Unsurprisingly, there were more guidelines on acute ischemic stroke than on ICH, and only a couple dedicated to cerebral venous sinus thrombosis (CVST). Comprehensive acute stroke guidelines were organized into pre-hospital, emergency setting, and hospital care sections. We followed the same approach to summarize the key recommendations in this document.

Overall, the concepts of the key recommendations were consistent across guidelines. However, we noticed some disagreements regarding the evaluation of the strength of the evidence supporting those recommendations. Such disagreements may be explained by different methods used for the grading of the evidence, but in some cases, a comparison of individual discussions of some topics (e.g. extended-window intravenous thrombolysis guided by perfusion imaging) indicated differences in how strong the evidence itself was considered to be.

Important knowledge gaps persist, and they are manifested by the lack of recommendations for some practical questions.

Of note, some individual guidelines had recommendations that differed from the rest. A notable example is the lower dose of alteplase in the Japanese guidelines (0.6 mg/kg).

### Main recommendations for secondary prevention

We identified 20 secondary stroke prevention topics for which there were strong recommendations, including the etiological diagnosis of ischemic stroke and TIA, lifestyle modification, management of specific risk factors, the use of antithrombotic agents for different cardioembolic sources, embolic stroke of undetermined source (ESUS), and extracranial/intracranial atherosclerotic disease, less frequent causes of ischemic stroke, and the prevention of recurrent spontaneous ICH. There were some differences between guidelines in their comprehensiveness and the strength and level of evidence assigned to specific recommendations. Strong recommendations for the secondary prevention of ICH were scarce.

Overall, the guidelines for the secondary prevention of ischemic stroke were comprehensive and covered all aspects of the continuum of care. However, there were no strong recommendations regarding some aspects of the secondary prevention of ICH because of the lack of robust evidence (e.g. specific blood pressure targets, the use of statins, and the management of antithrombotics post-ICH). Several ongoing randomized clinical trials will be concluded in the upcoming years and will likely contribute to address these knowledge gaps.

### Main recommendations for rehabilitation

Rehabilitation recommendations were extracted from guidelines about one particular problem (e.g. aphasia), all of rehabilitation/life after stroke, or the entire stroke pathway. None of the rehabilitation guidelines differentiated between ICH and ischemic stroke, implying that rehabilitation needs and interventions are equally effective for both stroke types. Whether this is the case is unknown, and is an area where further research is needed.

There were strong recommendations (Table 3) for organized stroke unit care, task-specific training, strength training, aerobic training, early supported discharge, goal setting, and avoiding very early mobilization. For other interventions, there was variation in strength of recommendations, for example, the VA group did not grade antidepressants for post-stroke depression as “strong” and the NICE guidelines (UK) included strong recommendations for rehabilitation of cognitive deficits, while other guidelines did not rate this as strong. One guideline^[Bibr bibr135-17474930231156753]^ recommended psychological treatment for anxiety (as in the general population), and one^[Bibr bibr202-17474930231156753]^ recommended selective serotonin reuptake inhibitor (SSRI), but we decided not to include these as the group judged that the underpinning evidence was insufficient. The variation in strength of recommendations might have reflected differences in the grading criteria, evidence included, and interpretation of evidence.

There are no strong recommendations for dysarthria, fatigue, apathy, and neglect. The only recommendation for end-of-life care was that palliative care services should be accessed.

### Quality of guidelines

The AGREE II tool demonstrated that guidelines generally did not consider resource implications or implementation. Thus, our subjective assessments of applicability had to be made based on own knowledge of resources in different health care settings. Ideally, services across the world should consider the cost-benefit of interventions based on local costs and resources available.

### Strengths of our approach

We published our protocol on PROSPERO; we followed this protocol except for the exclusion of pediatric guidelines because we did not have sufficient resources to evaluate those documents. We performed comprehensive searches, we convened an international group of experts in topic areas and systematic reviews with a balance of gender, seniority, and location.

### Weaknesses of our approach

To the best of our knowledge, there is no published methodology to synthesize the results of multiple guidelines. Although our approach was rigorous, some subjectivity, based on the judgment of the group members, was needed when guidelines differed in strength of recommendations for particular interventions.

There is no systematic way to our knowledge to investigate how the AGREE II tool can be used to identify which domain(s) influences recommendations. The previous WSO 2014 guidance took just two domains and selected 10 papers which had good scores in those two domains. Instead, we considered 200 publications identified in our searches. Further work is needed to develop ways to explore the influence of guideline quality on recommendations made. Researchers wishing to take this forward should contact us for access to the AGREE II assessments.

Although all the group was medically qualified and had broad range of experience from hyperacute care to rehabilitation and life after stroke, we did not have experts in other disciplines. We invited the WSO board members (which include stroke support organizations, with PWLE) to comment on our draft. We received several professional responses and lay responses. The next WSO guideline committee should seek to have more balanced professional/PWLE membership. This may require funding to enable PWLE to provide their time and expertise. Also, PWLE will be important in the dissemination stage of our work.

We did not search gray literature (i.e. literature that is not formally published in sources such as books or journal articles) or contact experts in the field to identify local/regional guidelines—due to insufficient resource. All the guidelines we reviewed were based on the same trials and systematic reviews, so it is unlikely that gray literature/regional guidelines would have come to different conclusions.

Ideally two authors would have extracted recommendations and reviewed quality for all guidelines, but this was not always feasible. It is unclear whether this would have improved the quality of our review process or changed our final recommendations.

Although we initially planned to exclude recommendation on TIA, we decided to modify the original PROSPERO protocol by including these recommendations. Reasons were that most recommendations for the prevention of ischemic stroke are also valid for TIAs and for specific recommendations, it would have resulted in reporting recommendations for stroke but not for TIA (e.g. dual antiplatelet therapy for minor stroke or TIA).

### How to use this information

This is the most comprehensive systematic evaluation of all world guidelines in stroke; however, the existence of guideline recommendations is meaningless without efforts to implement them in clinical practice. Services will need to consider the cost-benefits of different interventions in their own settings. Implementation of evidence is often challenging particularly for complex interventions that require changes in service structure and processes, or interventions for which new funding is needed. It is sometimes easier when disinvestment is needed or when simple changes are needed to organization of care. The recommendations we found generally require additional resources or reorganization of care pathways, although some of the recommendations require disinvestment (e.g. avoid very early mobilization). Based on the recommendations from this review, we then developed performance metrics for services, which may be used in audits (Table 4). These are at service level, process level, or individual patient level. A review of practice in line with findings in this review should be undertaken at multiple levels: individual clinicians, hospital teams/services, and across whole health systems.

### Implications for future guideline development and research

Guideline development is a resource-intensive process. Not only did we identify multiple superseded versions of guidelines, but there was duplication of work among different guideline groups, with the same trials and systematic reviews being cited in multiple guidelines. A central repository of stroke trials could be coordinated by WSO. Cochrane Stroke has maintained such a database for many years (Database of Research in Stroke (DORIS), www.askdoris.org/m), but with the disbanding of the UK Cochrane groups, DORIS will no longer be updated. However, with a modest investment, ongoing updates could be facilitated by the WSO. This will require further discussion.

Guideline groups should consider working more closely to avoid duplication such collaborative working could be coordinated by the WSO and focus on the evidence identification and data extraction stages. However, development and implementation of clinical recommendations must be contextualized for different health systems, and WSO should continue to support collaboration with guideline development groups.

To Guideline groups could also collaborate with a “living” guideline model.^208^ Living clinical guidelines ensure that evidence is kept up to date, as produced by the Stroke Foundation. Where there are common topic areas, groups could collaborate in the data extraction and overall evidence summaries. An often-cited potential risk to the living guideline approach is the possibility to change a recommendation every time a new trial is published. However, the Australian experience to date suggests this is rarely a consideration, with robust methods and strong clinical expert input, including period of public consultation to ensure new evidence is taken in context including consideration of other major trials underway which may impact the overall body of evidence.

We noted that guideline developers use different methods to grade the strength of recommendations. Guideline developers could consider using a common method to establish the strength of recommendations, such as GRADE.

There were important gaps—some problems of importance to patients (e.g. fatigue) were not featured, largely due to the lack of primary research. For rehabilitation, there was an implicit assumption that the pathology of the stroke lesion is not relevant to choices about rehabilitation. Future guideline groups should define “consider.” Lay membership influences the conduct of guideline development, scope, inclusion of patient-relevant topics, outcome selection, and planned approaches to recommendation development, implementation, and dissemination with implications for both guideline developers and the guideline development process; therefore, lay members should be embedded within guideline development.^209^ Also, a more robust assessment of resource implications and implementation is needed. Finally, the burden of comorbidity is increasing with population aging, and the majority of current guidelines did not specifically address the impact of comorbidity, frailty, and polypharmacy on recommendations. This needs to be addressed by researchers and guideline developers in the future to ensure that evidence is applicable to the patients we treat.

### WSO guidelines committee membership

Co-chairs: Gillian E Mead and Alejandro Rabinstein

Leads of subgroups: Acute care: Alejandro Rabinstein and Gisele Sampaio Silva; Secondary prevention: Luciano Sposato and Kolawale Wahab; Rehabilitation and life after stroke: Gillian Mead and Laetitia Yperzeele

Members: Patrice Lindsay, PN Sylaja, Vijay Kumar Sharma, Gisele Sampaio Silver, Mansur Kutlubaev, Simiao Wu, Mary Kay Ballastiotes (PWLE), Kelvin Hill, Victor Urrutia, Pooja Khatri, Anil Yalaprada, David Liebeskind, Mayowa Owolabi.

## Supplemental Material

sj-docx-1-wso-10.1177_17474930231156753 – Supplemental material for A systematic review and synthesis of global stroke guidelines on behalf of the World Stroke Organization Click here for additional data file. Supplemental material, sj-docx-1-wso-10.1177_17474930231156753 for A systematic review and synthesis of global stroke guidelines on behalf of the World Stroke Organization by Gillian E Mead, Luciano A Sposato, Gisele Sampaio Silva, Laetitia Yperzeele, Simiao Wu, Mansur Kutlubaev, Joshua Cheyne, Kolawole Wahab, Victor C Urrutia, Vijay K Sharma, PN Sylaja, Kelvin Hill, Thorsten Steiner, David S Liebeskind and Alejandro A Rabinstein in International Journal of Stroke
