# Supplementary material for: A systematic review and synthesis of global stroke guidelines on behalf of the World Stroke Organization
Source: Int J Stroke. 2023 Mar 1;18(5):499–531. doi: 10.1177/17474930231156753 (PMC10196933; doi:10.1177/17474930231156753)
Supplement: sj-docx-1-wso-10.1177_17474930231156753 – Supplemental material for A systematic review and synthesis of global stroke guidelines on behalf of the World Stroke Organization [file sj-docx-1-wso-10.1177_17474930231156753.docx]

**Appendix 1**

Search methods

We searched for guidelines published since 1st January 2011 in any language using the following resources:

DORIS (Database of Research in Stroke; [www.askdoris.org/](about:blank))

Cochrane Register of Studies (CRS; [www.community.cochrane.org/help/tools-and-software/crs-cochrane-register-studies/about-crs](about:blank))

MEDLINE Ovid (from 1946) (Appendix/ Supplementary 1);

Embase Ovid (from 1974);

TRIP (Turning Research into Practice) Database via 2dSearch ([www.2dsearch.com](about:blank))

Google via 2dSearch (www.2dsearch.com/)

The subject search strategy was adapted from an existing search on the MEDLINE Ovid platform designed to retrieve all potential references in stroke healthcare and medicine (Cheyne 2020). The search strategy uses a combination of Medical Subject Headings (MeSH) from the National Library of Medicine, uncontrolled vocabulary/keywords in the Text Word (.tw) field, and Boolean operators to maximise sensitivity and recall. This search was combined with a search strategy designed to retrieve guidelines. This search was used as the basis for all additional searches.

These primary searches were supplemented by a search of the Guideline folder within the Cochrane Register of Studies (CRS), a ‘meta-register’ of Specialised Registers from all Cochrane groups of all the trials identified by Cochrane is populated by Anne Eisinga, Information Specialist with the Cochrane UK core staff in Oxford, UK.

We supplemented this with searches of TRIP, MEDLINE/PubMed and 2dSearch.

We had intended to contact experts and guidelines development groups worldwide to help locate additional relevant national and regional guidelines, including all major stroke organizations affiliated with the WSO, but this was no feasible due to the large number of guidelines identified.

URL to search strategy. Cheyne, JD. (2020). Search strategy for retrieval of references on stroke healthcare in MEDLINE Ovid, [text]. University of Edinburgh. College of Medicine and Veterinary Medicine. Cochrane Stroke Group. [https://doi.org/10.7488/ds/2862](about:blank)

**Appendix 2**

Results of systematic searches

Systematic Searches

Searches were performed (JC) on 6^th^ September 2021 (see PRISMA), 1540 references imported into COVIDENCE on 8^th^ September 2021, 1351 duplicates removed, 14049 citations scrutinised, 13138 irrelevant citations excluded; 911 full texts were reviewed, 219 articles were selected for retrieval as full texts. An additional six were identified from other sources including four from stakeholder review (one after the date of our searches and three prior to search date). These publications did not result in a change in our recommendations. We included 200 guidelines after excluding duplicates.

Exclusion of further full texts

Nine guidelines that covered only paediatric stroke were excluded as we did not have the resource to review these [22, 23, 24, 88, 103, 111, 112, 123, 193].

Another 54 publications were also excluded by the individual subgroups (Appendix 3). Note that some guidelines which were excluded by one group because they were out of scope were still used if relevant by other groups.

**Appendix 3**

Publications excluded after allocation to the guidelines to the three subgroups

|  | **Acute group (citations in bold refer to those in the list of references)** | **Secondary prevention group** | **Rehabilitation group** |
| --- | --- | --- | --- |
| Does not fulfil definition of a guideline about management of stroke | **120,** **132, 192 178** | **Not a secondary prevention guideline**: **41, 52,** **75, 78, 114, 115, 120, 145, 131, 186, 192** | **1, 34, 92** |
| Superseded version | **8, 35**, **36, 50, 52, 54, 75, 94, 99, 131, 143, 157, 162, 163** | **58, 59, 82**, **98, 102, 125, 129, 130, 139, 153, 164, 170, 168, 185, 187** | **27** |
| In a language not spoken by the members of the relevant subgroup |  | **40 (Croatian), 53 (Chinese), 66 (German), 67 (German), 195 (Chinese)** |  |
| Other reasons for exclusion |  | **154** Not available |  |
| TOTAL number excluded | **18** | **32** | **4** |

**Appendix 4**

**Contraindications for Intravenous Thrombolysis**

| Head CT showing acute intracranial hemorrhage or large acute-appearing hypodensity in the expected ischaemic territory |
| --- |
| Severe acute head trauma |
| Acute aortic dissection |
| Suspected aneurysmal subarachnoid hemorrhage |
| Suspected infective endocarditis |
| History of intracranial hemorrhage |
| Active gastrointestinal malignancy with high bleeding risk |
| Intra-axial central nervous system tumor |
| History within the previous 3 months of one of the following:  Severe head trauma or severe ischaemic stroke  Intracranial or intraspinal surgery  Gastrointestinal bleeding |
| Coagulopathy  Platelets <100,000 per mm3  INR >1.7  PT >15 sec  aPTT >40 sec  Therapeutic dose of LMWH within previous 24 hours |
